# Factors Associated with the Usefulness of Public Health Communication in the Context of COVID-19: Lessons Learned from the African, Caribbean, and Black Communities in Ottawa, Ontario

**DOI:** 10.3390/idr15050051

**Published:** 2023-09-07

**Authors:** Josephine Etowa, Bishwajit Ghose, Egbe Etowa, Charles Dabone, Malemo Luc, Amoy Jacques, Susan Roelofs, Ubabuko Unachukwu, Danielle Brown-Shreves, Glory Osandatuwa, Haoua Inoua

**Affiliations:** 1 School of Nursing, Faculty of Health Sciences, University of Ottawa, Ottawa, ON K1A 0A1, Canada; 2 Interdisciplinary School of Health Sciences, Faculty of Health Sciences, University of Ottawa, Ottawa, ON K1A 0A1, Canada; 3 Daphne Cockwell School of Nursing, Faculty of Community Services, Toronto Metropolitan University, Toronto, ON M5B 2K3, Canada; 4 Canadians of African Descent Health Organization, Ottawa, ON K1H 8M5, Canada; 5 Ottawa Public Health, Ottawa, ON K2G 6J8, Canada; 6 CO-CREATH Lab, Faculty of Health Sciences, University of Ottawa, Ottawa, ON K1A 0A1, Canada; 7 C.T. Lamont Primary Care Research Centre, Bruyère Research Institute, Ottawa, ON K1R 6M1, Canada; 8 Department of Family Medicine, Queens University, Kingston, ON K7L 3G2, Canada; 9 Faculty of Medicine, University of Ottawa, Ottawa, ON K1H 8M5, Canada; 10 Restore Medical Clinics, Ottawa, ON K1S 4G4, Canada; 11 AIDS Committee of Ottawa, Ottawa, ON K1S 1A9, Canada

**Keywords:** public health messages, COVID-19, African, Caribbean, and Black, Ottawa, Ontario

## Abstract

Public health communication is critical for promoting behaviours that can prevent the transmission of COVID-19. However, there are concerns about the effectiveness of public health communication within Canada’s African, Caribbean, and Black (ACB) communities. In the community sample of ACB people in Ottawa, Ontario, we asked community members if they perceive public health message related to COVID-19 to be effective. Using this question, the current study aimed to explore factors associated with the perceived usefulness of public health messages related to COVID-19. Results from the multivariate analysis have shown that ACB people with lower levels of risk perception for COVID-19 were less likely to perceive that public health messages were useful (OR = 0.405, *p* < 0.01). In addition, mistrust in government COVID-19 information was also negatively associated with their perception that health messages are useful (OR = 0.169, *p* < 0.01). For socioeconomic status, ACB people with no high school diploma (OR = 0.362, *p* < 0.05) and income dissatisfaction (OR = 0.431, *p* < 0.05) were less likely to report the perceived usefulness compared to those with a bachelor’s degree and income satisfaction. Based on these findings, we discussed implications for policymakers and directions for future research.

## 1. Introduction

The COVID-19 pandemic has brought unprecedented challenges to public health systems worldwide. Governments and public health organizations have been issuing a range of recommendations and guidelines to mitigate the spread of the virus, such as reducing physical contact, washing hands, wearing masks, and getting vaccinated [1]. Research has demonstrated that among the multifaceted factors considered to promote behaviors that prevent COVID-19 transmission, public health communication emerges as one of the critical structural determinants [2]. Yet, the effectiveness of these measures largely depends on the perceived usefulness of public health communication, considering that individuals are more likely to act on it when they perceive a message to be useful [2]. Therefore, it is vital to ensure that public health messages are perceived as useful and relevant to enable and motivate individuals to adopt the intended COVID-19 recommendations conveyed by these messages [3].

Despite the importance of public health messages, research shows that Canada’s African, Caribbean, and Black (ACB) communities face unique barriers to effective public health communication [4,5]. For example, the ACB communities may have challenges trusting health information from the government or healthcare providers due to historical and ongoing experiences of discrimination, bias, and mistreatment [6]. Moreover, cultural and linguistic characteristics can serve as barriers to effective communication among the ACB communities as these messages may not be tailored to meet their unique differences [7]. Furthermore, the ACB communities experience structural socioeconomic inequalities, which can limit their access to healthcare information and resources [8].

Prior research has identified a complex interplay of psychosocial, demographic, and socioeconomic factors that may be impacting the perceived usefulness of public health communication in the context of COVID-19 [9]. For example, COVID-19 knowledge, risk perception, prior vaccine experience, and attitudes toward COVID-19 media messages have been identified as possible underlying psychosocial factors that may be associated with effective public health communication [10,11]. In addition, some studies have shown that younger individuals and women are more likely to engage in COVID-19 preventive behaviours in response to public health messages, pointing to the importance of considering demographic factors such as age and gender [12,13]. Socioeconomic factors such as income and education have been associated with the effectiveness of public health messages on COVID-19, indicating that people with lower income and educational levels were more likely to face barriers to accessing and understanding these messages [14].

Although these findings are important, very few studies have examined the factors associated with the usefulness of public health messages related to COVID-19 among ACB people in Canada. This void in the literature is particularly concerning because ACB people in Canada have been disproportionately impacted by COVID-19 [6,8]. A number of studies have shown that ACB people are more likely to report COVID-19 vaccine hesitancy compared to their white counterparts, even after accounting for possible confounding factors such as demographic and socioeconomic factors [15,16,17]. Similarly, based on the Canadian Community Health Survey collected in 2021 and 2022, 82% of Black people received at least one dose of a COVID-19 vaccine, while this figure was higher for individuals who are neither Indigenous nor part of visible minorities (93%). In addition, research has shown that the ACB communities have experienced a higher burden of COVID-19 cases, hospitalizations, and deaths compared to other racial/ethnic groups [18]. These findings may highlight the importance of understanding whether ACB people view public health communication related to COVID-19 as useful and relevant as well as what factors are associated with their views. To this end, the current study aims to advance the literature by identifying the psychosocial, demographic, and socioeconomic correlates of the usefulness of public health messages related to COVID-19 among ACB people in Ottawa, Ontario, Canada.

## 2. Materials and Methods

### 2.1. Data Source

This study used the data from the ACB Vaccine Acceptance Project. More about this project and dataset can be found elsewhere [19].

### 2.2. Measures

Respondents were asked whether the COVID-19-related messages are useful to prevent community-level infection. Based on this information, we constructed the dependent variable: ‘perceived usefulness of public health messages related to COVID-19’. There were three blocks of independent variables in this study—including psychosocial (i.e., level of COVID-19 knowledge, ACB population has a higher risk, vaccinated for any non-COVID-19 diseases, and trust information from the government), demographic (i.e., language, marital status, age of respondents, gender, and citizenship status), and socioeconomic factors (i.e., education and income satisfaction). A coding strategy for each independent variable has been shown in Table 1 and Table 2.

### 2.3. Statistical Analysis

Logistic regression analysis was fitted to explore the factors associated with the usefulness of public health messages related to COVID-19 among ACB people in Canada [20]. There were two important steps to logistic regression analysis. First, we estimated the binary relationship between the usefulness of public health messages related to COVID-19 and three sets of explanatory variables. Second, we further conducted multivariate statistics to generate net estimates. Through the utilization of the ipfweight command in Stata [21], we further employed the iterative proportional fitting (IPF) technique to enhance the comparability of the survey sample with population estimates obtained from the 2016 Census data (considering that the 2021 Census data have not been publicly available). As shown in Table 1, we observed that several demographic characteristics of our sample were largely different from those at the population level, particularly including marital status (i.e., formerly married—9% vs. 15%; never married—38% vs. 43%; currently married—53% vs. 42%), citizenship status (i.e., 85% vs. 78%; 15% vs. 22%), and education (i.e., bachelor’s degree or higher—55% vs. 27%; high school or some college—35% vs. 61%). By leveraging these characteristics, the IPF process facilitated the adjustment of survey weights, iteratively refining them until convergence was achieved. This iterative approach allowed for the systematic alignment of the sample distribution with the known population parameters, reducing potential bias and improving the overall representation of the target population. Sampling weights generated from this procedure were applied to regression analysis. In all analyses, *p* < 0.05 was set as a cut-off for statistical significance, due to the relatively small nature of the sample size. All analyses were conducted using Stata 15 (StataCorp, College Station, TX, USA).

## 3. Results

Table 2 presents sample characteristics. We found that 64% of ACB people from the sample in Ottawa believe that messages related to COVID-19 are useful to prevent community-level infection. In addition, 30% believe that the ACB population is at a higher risk for COVID-19 compared to other racial/ethnic groups, while 74% have received at least one dose of any non-COVID-19 vaccine. Furthermore, 55% of respondents reported that they trust COVID-19 information from the government.

Table 3 presents bivariate findings, which show that various psychosocial, demographic, and socioeconomic factors are associated with the perceived usefulness of public health messages related to COVID-19 among ACB people. At the bivariate level, we found that individuals with a low level of COVID-19 knowledge were less likely to perceive public health messages as useful compared to those with a high level of knowledge (OR = 0.472, 95% CI = 0.257, 0.866). Similarly, those who did not perceive ACB populations to be at higher risk for COVID-19 were less likely to find public health messages useful (OR = 0.457, 95% CI = 0.249, 0.839). Additionally, respondents who did not trust government reports on COVID-19 were less likely to perceive public health messages as useful (OR = 0.172, 95% CI = 0.101, 0.294). In terms of demographic factors, we found that respondents aged 55+ were less likely to perceive public health messages as useful, compared to those aged 18–34 (OR = 0.442, 95% CI = 0.214, 0.913). Regarding socioeconomic factors, those with less than a high school education (OR = 0.366, 95% CI = 0.171, 0.783) as well as neutral (OR = 0.481, 95% CI = 0.261, 0.886) and dissatisfied income (OR = 0.536, 95% CI = 0.288, 0.994) were less likely to perceive public health messages as useful compared to those with a bachelor’s degree or higher and income satisfaction.

Table 3 also shows multivariate findings, which were largely consistent with bivariate findings. We found that individuals with a low level of COVID-19 knowledge were less likely to perceive public health messages as useful compared to those with a high level of knowledge (OR = 0.462, 95% CI = 0.231, 0.923). We also found that those who did not perceive ACB populations to be at higher risk for COVID-19 were less likely to find public health messages useful (OR = 0.405, 95% CI = 0.210, 0.780). Also, respondents who did not trust government reports on COVID-19 were less likely to perceive public health messages as useful (OR = 0.169, 95% CI = 0.094, 0.304). Consistent with bivariate findings, those with less than a high school education (OR = 0.362, 95% CI = 0.138, 0.949) and income dissatisfaction (OR = 0.431, 95% CI = 0.218, 0.856) were less likely to perceive public health messages as useful, compared to those with a bachelor’s degree or higher and income satisfaction.

## 4. Discussion

Effective public health communication is crucial for promoting behaviours that prevent the transmission of COVID-19. However, there may be unique challenges to effective communication among vulnerable groups, such as the ACB communities in Canada. This is especially important since the ACB communities have been disproportionately affected by COVID-19, with higher infection and mortality rates compared to other groups. To address this issue, this study aims to identify psychosocial, demographic, and socioeconomic factors associated with the perceived usefulness of public health messages related to COVID-19 among ACB community members in Ottawa, ON, Canada.

As part of psychosocial factors, for example, we found that higher levels of community-level risk perception related to COVID-19 were associated with the perceived usefulness of public health messages among ACB people. This finding is consistent with previous research in the United States [22], showing that individuals with higher levels of risk of COVID-19 were more likely to view health messages as personally relevant, leading to greater message acceptance and behavioural intention to comply with health recommendations. Additionally, higher levels of perceived risk were associated with greater motivation to seek health information related to COVID-19 in Italy, potentially increasing their appreciation and acceptance of acquired information [23]. It is possible that the recognition of unique vulnerabilities that ACB people face systematically may have impacted their perception of the usefulness of public health messages related to COVID-19. 

Furthermore, trust in information related to COVID-19 from government reports was another psychosocial factor associated with the perceived usefulness of public health messages among members of the ACB community. This finding aligns with previous research in Canada [24], indicating the importance of trust in government institutions and health authorities in accepting health communication as legitimate and useful. However, it is crucial to contextualize these findings within the historical and ongoing experiences of discrimination faced by ACB people in Canada [25,26]. Discrimination in various forms, including within the healthcare system, can erode trust in government institutions, including public health officials and their information about COVID-19 [5,6]. In this context, some members of the ACB community may view government information as biased and incomplete, potentially contributing to lower levels of perceived usefulness of public health messages related to COVID-19.

Our study revealed that ACB individuals without a high school diploma and those who reported income dissatisfaction were less likely to perceive public health messages as useful. This finding may be explained by findings that low income is often associated with limited access to resources such as healthcare facilities, preventive measures, and health information in Canada [27,28]. Low income is also concentrated among ACB essential workers who often had to work in jobs that require them to interact in close proximity to others during the pandemic, being exposed to a higher risk of contracting COVID-19 [29,30]. In this context, public health messages may be perceived to be less relevant and useful among those facing financial difficulties. 

Education also plays a critical role in improving health literacy and critical thinking skills, enabling individuals to better understand and evaluate public health messages. It has been suggested that people with higher levels of education have better knowledge of COVID-19 and are more likely to engage in preventive measures such as wearing masks, washing hands, and getting vaccinated [14]. Reflecting on this result, understanding the root causes of health and related behavioural disparities may require essential contextualization within the persistent systemic discrimination in education and employment that ACB communities continue to face in Canada [31]. The Canadian Race Relations Foundation [32] has noted that Black Canadians are less likely to have post-secondary education than non-Black Canadians and are overrepresented in precarious low-wage jobs.

## 5. Conclusions

Based on these findings, we have several policy implications. First, to address the psychosocial impact on the perceived usefulness of health communication, policymakers may need to address issues such as lower COVID-19 risk perception and mistrust of government COVID-19 information. Specifically, policymakers should focus on establishing culturally tailored communication strategies that integrate diverse cultural backgrounds and languages within ACB communities. Policymakers may enhance engagement and foster trust between the government and ACB communities through (1) providing information in a culturally sensitive manner, (2) utilizing appropriate languages, and (3) incorporating community-specific messaging that acknowledges past and ongoing experiences of discrimination and injustices. During this process, collaboration with trusted community leaders may also be essential in co-creating and disseminating accurate information, leveraging their networks and influence to bolster trust in government information. Second, policymakers may continue to address systemic disparities faced by ACB communities in areas such as education, employment, and income, considering the concentration of low-income earners among essential workers in these communities. In this context, policymakers should organize community-based educational sessions and workshops where healthcare professionals directly engage with ACB communities. These initiatives may effectively bridge the educational inequality gap by disseminating accurate information, addressing concerns, and fostering a participatory and community-centric approach to building trust in government information.

While these policy recommendations may be useful, it is important to acknowledge the limitations of this study. The cross-sectional survey design used in this study restricts the ability to determine the temporal order of variables and establish causal relationships. Therefore, our results are limited to identifying statistical associations only. In addition, social desirability bias may have influenced the reported perceived usefulness of public health messages, as respondents may overreport their perceived usefulness due to societal expectations. Furthermore, this study was conducted using a community sample of ACB individuals in Ottawa, and thus the findings may not be applicable to other ACB communities as well as different ethnic and racial minority groups in Canada. In light of the survey’s relatively low response rate, it is pertinent to consider augmenting the sample size, possibly through the execution of multisite research initiatives focused on COVID-19 vaccine perceptions among ACB individuals in Canada. To address these limitations, future studies should consider using mixed method approaches that integrate both longitudinal quantitative techniques and in-depth qualitative interviews and observations at the national level. Such an approach may be useful in capturing the unique decision-making processes that ACB and other racial and ethnic minority populations undergo in shaping their perceived usefulness of public health messages. Despite the limitations of our study, it is important to note that our investigation contributes to the relatively limited body of research examining the determinants of the effectiveness of public health communication among ACB communities. Our findings were largely consistent with outcomes from prior research conducted with ACB communities and beyond in many other jurisdictions.

## Figures and Tables

**Table 1:** Sample characteristics.

	Sample Characteristics	Population Characteristics
**Age**		
55+	18	17
35–54	41	40
18–34	41	42
**Gender**		
Female	58	57
Male	42	43
**Marital status**		
Formerly married	9	15
Never married	38	43
Currently married	53	42
**Citizenship status**		
Citizen	85	78
Non-citizen	15	22
**Education**		
Bachelor’s degree or higher	55	27
High school or some college	35	61
Less than high school	10	12

**Table 2:** Questionnaire responses.

	Percentage
**Perceived usefulness of public health messages related to COVID-19**	
No	36
Yes	64
**COVID-19 knowledge**	
High	36
Middle	31
Low	33
**ACB communities have a higher risk**	
Yes	30
No	70
**Vaccinated for any non-COVID-19 diseases**	
Yes	74
No	26
**Trust COVID-19 information from the government**	
Yes	55
No	45
Total	375

**Table 3:** Logistic regression analyses predicting whether respondents agree that messages related to COVID-19 are useful to prevent community-level infection.

	Unadjusted			Adjusted		
	OR	95% CI		OR	95% CI	
**Level of COVID-19 knowledge**						
Low	1.000			1.000		
Middle	0.797	0.415	1.530	0.892	0.461	1.726
High	0.472 **	0.257	0.866	0.462 **	0.231	0.923
**ACB population has a higher risk**						
Yes	1.000			1.000		
No	0.457 **	0.249	0.839	0.405 ***	0.210	0.780
**Vaccinated for any non-COVID-19 diseases**						
Yes	1.000			1.000		
No	0.591	0.338	1.031	0.830	0.498	1.382
**Trust information from the government**						
Yes	1.000			1.000		
No	0.172 ***	0.101	0.294	0.169 ***	0.094	0.304
**Language**						
English	1.000			1.000		
French	0.874	0.527	1.449	0.677	0.386	1.186
**Marital status**						
Formerly married	1.000			1.000		
Never married	0.914	0.444	1.887	0.915	0.438	1.908
Currently married	0.599	0.311	1.154	0.575	0.286	1.157
**Age of respondents**						
18–34	1.000			1.000		
35–54	0.681	0.325	1.427	0.840	0.369	1.917
55+	0.442 **	0.214	0.913	0.830	0.355	1.939
**Gender**						
Female	1.000			1.000		
Male	0.657	0.393	1.099	0.953	0.520	1.748
**Citizenship status**						
Citizen	1.000			1.000		
Non-citizen	0.763	0.382	1.521	1.342	0.587	3.068
**Education**						
Bachelor’s degree or higher	1.000			1.000		
High school or some college	0.739	0.458	1.194	0.815	0.464	1.431
Less than high school	0.366 **	0.171	0.783	0.362 **	0.138	0.949
**Income satisfaction**						
Satisfied	1.000			1.000		
Neutral	0.481 **	0.261	0.886	0.624	0.324	1.201
Dissatisfied	0.536 **	0.288	0.994	0.431 **	0.218	0.856

** *p* < 0.05, *** *p* < 0.01; OR = odds ratio; CI = confidence intervals.

## Data Availability

The data presented in this study are available on request from the corresponding author. The data are not publicly available due to privacy and ethical restrictions.
